# Thermal Actuation Based 3-DoF Non-Resonant Microgyroscope Using MetalMUMPs

**DOI:** 10.3390/s90402389

**Published:** 2009-04-01

**Authors:** Rana Iqtidar Shakoor, Shafaat Ahmed Bazaz, Michael Kraft, Yongjun Lai, Muhammad Masood ul Hassan

**Affiliations:** 1 Department of Chemical and Materials Engineering, Pakistan Institute of Engineering and Applied Sciences, Islamabad, Pakistan; 2 Faculty of Electronics Engineering, GIK Institute of Engineering and Applied Sciences, Topi, NWFP, Pakistan; 3 School of Electronics and Computer Science, Southampton University, Highfield, Bld 86, 2033, Southampton, UK, SO17 1BJ; 4 Department of Mechanical Engineering, Queens University, Kingston, ON, Canada, K7L 3N6

**Keywords:** Finite element method, micromachined gyroscope, MEMS, thermal V shaped actuator, chevron-shaped actuator

## Abstract

High force, large displacement and low voltage consumption are a primary concern for microgyroscopes. The chevron-shaped thermal actuators are unique in terms of high force generation combined with the large displacements at a low operating voltage in comparison with traditional electrostatic actuators. A Nickel based 3-DoF micromachined gyroscope comprising 2-DoF drive mode and 1-DoF sense mode oscillator utilizing the chevron-shaped thermal actuators is presented here. Analytical derivations and finite element simulations are carried out to predict the performance of the proposed device using the thermo-physical properties of electroplated nickel. The device sensitivity is improved by utilizing the dynamical amplification of the oscillation in 2-DoF drive mode using an active-passive mass configuration. A comprehensive theoretical description, dynamics and mechanical design considerations of the proposed gyroscopes model are discussed in detail. Parametric optimization of gyroscope, its prototype modeling and fabrication using MetalMUMPs has also been investigated. Dynamic transient simulation results predicted that the sense mass of the proposed device achieved a drive displacement of 4.1μm when a sinusoidal voltage of 0.5V is applied at 1.77 kHz exhibiting a mechanical sensitivity of 1.7μm /°/s in vacuum. The wide bandwidth frequency response of the 2-DoF drive mode oscillator consists of two resonant peaks and a flat region of 2.11 kHz between the peaks defining the operational frequency region. The sense mode resonant frequency can lie anywhere within this region and therefore the amplitude of the response is insensitive to structural parameter variations, enhancing device robustness against such variations. The proposed device has a size of 2.2 × 2.6 mm^2^, almost one third in comparison with existing M-DoF vibratory gyroscope with an estimated power consumption of 0.26 Watts. These predicted results illustrate that the chevron-shaped thermal actuator has a large voltage-stroke ratio shifting the paradigm in MEMS gyroscope design from the traditional interdigitated comb drive electrostatic actuator. These actuators have low damping compared to electrostatic comb drive actuators which may result in high quality factor microgyroscopes operating at atmospheric pressure.

## 1. Introduction

Micromachining has made possible the production of precision inertial sensors at a price that allows their usage in cost-sensitive consumer applications. Conventional rotating wheel as well as precision fiber-optic and ring laser gyroscopes all are too expensive and too large for use in most emerging applications. Micromachining can shrink the sensor size by orders of magnitude, reduce the fabrication cost significantly, and allow the electronics to be integrated on the same silicon chip [1].

Practically all MEMS gyroscopes use the Coriolis Effect; a proof mass is driven into oscillation (the drive mode) and in the presence of a rotational motion the proof mass starts to oscillate in the sense mode if sense and drive mode directions and the rotational axis are mutually perpendicular. During the development of micromachined gyroscopes, various actuation mechanisms have been explored to oscillate the vibrating structure in the primary drive mode; the most common ones include electrostatic, piezoelectric and electromagnetic means [2–5]. Electrostatic actuation using a comb drive design is the currently prevailing approach [2–3] as they require a low voltage and can excite high frequency resonant modes. However, the resulting deflection amplitude is relatively small. To detect the Coriolis-induced vibrations in the secondary sense mode, capacitive, piezoresistive or piezoelectric pick-off mechanisms are commonly used.

During last couple of years, extensive research has been carried out on actuators using thermal expansion effects [6–10]. These actuators are activated by Joule heating. When a potential difference is applied between two or more points on an electrically conducting, elastic continuum it causes a flow of electrical current which induces thermal strain due to internal heating. Consequently, this thermal strain generates the desired mechanical deformation. Topology and shape of the continuum give rise to non-uniform joule heating and hence non-uniform thermal expansion [7]. These thermal actuators can provide a large force and actuation both in parallel and perpendicular to the substrate and maybe fabricated using surface-micromachining technology that is compatible with IC technology.

Two types of thermal actuators are very common: hot/cold arm thermal actuators and ‘V’ or ‘chevron-shaped’ actuators.

**Figure 1.**
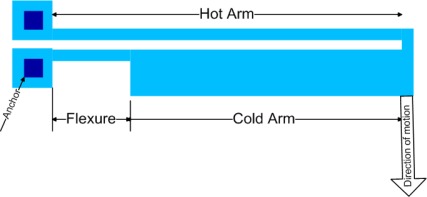
Hot/cold arm thermal actuator.

The hot/cold arm thermal actuator as shown in Figure 1 consists of a long and thin hot arm and a wider cold arm connected at one end through a short thin flexure [6–9]. A potential difference across the two anchors causes a current flow from the hot arm to a flexure via a cold arm. This results in a higher current density in both the hot arm and the flexure than in the cold arm and thus both of these will heat more than the cold arm. The net expansion creates a moment that bends the entire structure.

**Figure 2.**
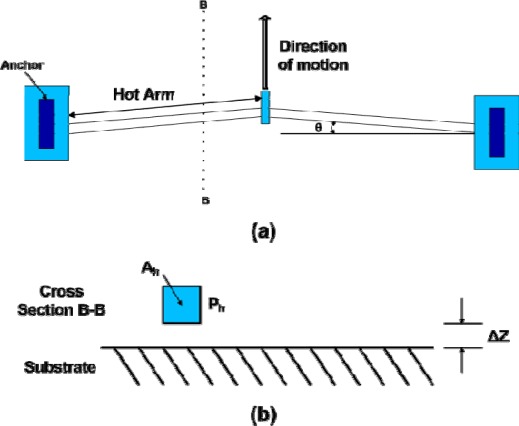
Schematic of a chevron-shaped thermal actuator (a) top view (b) cross section B-B view.

The chevron-shaped thermal actuator as shown in Figure 2 consists of two thin hot arms at a small angle *θ* with respect to each other. This is a toggle mechanism that provides large motion amplification, proportional to 1/*θ* for small angles. The displacement is proportional to the square of the applied voltage and maximum displacement is limited by buckling of the hot arm at high temperature [6, 9 and 10].

Comparing with hot/cold arm actuators, the chevron-shaped actuators produce more force which can be increased by multiple pairs of hot arms in parallel [6]. The chevron-shaped actuators are more efficient than hot/cold arm actuators as there is no cold arm to reduce the net expansion.

In this paper we presented a novel Nickel based 3-DOF non-resonant micromachined vibratory gyroscope which utilizes a chevron-shaped thermal actuator for driving the vibrating proof mass of the gyroscope in the primary drive mode. The main motivation to use a chevron-shaped thermal actuator instead of a conventional electrostatic actuator is its distinctiveness in terms of high force generation combined with the large displacements at a low excitation voltage. Furthermore, such actuators may enable higher quality factor compared to the comb drive actuators, as it reduce the damping significantly and enhancing the use of such chevron-based gyroscopes at atmospheric pressure. In fact, high force, large displacement and low voltage consumption are a primary concern for microgyroscopes. Electroplated Nickel was used as the structural layer for this chevron-based microgyroscope since metals are much better for such heat actuators as they provide a relatively large deflection and force for low operating temperatures and power consumption. The lateral deflection of the heat actuators made from Ni metal is about ∼ 60% larger than that of the Si based actuators under the same power consumption [11]. The simulated results presented in this study predict that the chevron-shaped actuators made from the metal may have very promising characteristics for the drive mode actuation of microgyroscopes.

In this paper, Section 2 describes the background theory of the non-resonant design approach with a 2-DOF drive and 1-DoF sense mode oscillators along with suspension design implementation for the proposed gyroscope. Section 3 includes parametric optimization methodology for the proposed gyroscope with the brief description of the MetalMUMPs process and prototype modeling of the device. Section 4 cover an FEM based systematic sequential thermoelectromechanical analysis methodology for the proposed gyroscope using the MEMS design software IntelliSuite. In the final section, simulation results for modal, static and dynamic transient analyses of proposed microgyroscope are presented.

## 2. Design concept and its Implementation

### 2.1. Non-resonant 3-DoF micromachined vibratory gyroscopes (MVG)

In 2-DoF resonant micromachined vibratory gyroscopes, a single proof mass is anchored above the substrate through flexible suspensions. The proof mass is driven into resonance by an external sinusoidal force which is generated by applying a voltage to the comb drive structure. When the gyroscope is subjected to an angular rotation, Coriolis force is induced in the sense direction. The general expression for the Coriolis force is *F⃗_C_* = 2*m*Ω⃗ × *v⃗* where *v⃗* is the translational velocity of the proof mass in the rotating system, and Ω⃗ is the angular velocity vector. If both the drive and sense mode have the same resonant frequencies, the Coriolis force excites the system into resonance in the sense direction.

However, fabrication tolerances and environmental changes drastically affect the suspension stiffness which causes resonant mode mismatch between the sense and drive modes. Furthermore, slight asymmetries in the structure due to fabrication imperfections cause both anisoelasticity in the gyroscopes structure and dynamic cross-coupling between the drive and sense directions. All these factors play a major role in limiting the performance of resonant gyroscopes [13–15].

For the last several years, research has being carried out to enhance the inherent robustness of micromachined vibratory rate gyroscopes against structural and environmental parameter variations. Wide bandwidth frequency responses in drive and sense-modes play a vital role in enhancing this robustness. A dynamic system with a wide-bandwidth frequency response can be achieved either by increasing the degree-of-freedom (DoF) of the drive and sense mode oscillatory system or by utilizing multiple drive-mode oscillators with incrementally spaced resonant frequencies [16]. Therefore, an approach using a 3-DoF dynamic system has been utilized in contrast to conventional 2-DoF resonant MVG to increase the bandwidth of the proposed thermally actuated vibratory rate gyroscope.

**Figure 4.**
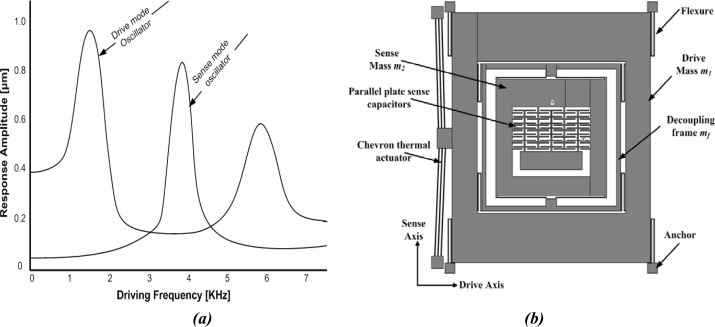
(a) 3-DoF design concept with 2-DoF drive and 1-DoF sense mode oscillators. (b) Proposed Gyroscope model with 2-DoF Drive mode and 1-DoF sense mode oscillator.

This design approach aims to utilize resonance either in the drive mode or sense mode, but not in both, to improve sensitivity while maintaining the robust operational characteristics. This can be achieved, for example, by designing structurally decoupled 1-DoF and 2-DoF oscillators in the sense and drive-modes respectively. These will result in two resonance peaks in the frequency response of the 2-DoF drive-mode oscillator with a flat region between both peaks which defines the operational frequency region. The resonance frequency of 1-DoF sense mode oscillator is designed in such a way to lie within the flat frequency region of the drive-mode eliminating the requirement of mode matching in comparison to conventional 2-DoF resonance microgyroscopes. This is shown in figure 4a. Thus the device is operated at the resonance frequency of 1-DoF oscillator, while the 2-DoF oscillator amplitude is inherently constant within the operational frequency band.

The overall 3-DoF micromachined vibratory gyroscopes consist of two interconnected proof masses *m_1_* and *m_2_* shown in Figure 4*(b)*. Mass *m_1_* is excited in the drive direction (X-Axis) whereas it is constrained to oscillate in the sense direction. The chevron-shaped thermal actuators are being used for driving the gyroscope drive masses. Mass *m_2_* can oscillate both in the drive and sense directions (Y-Axis). In this way the gyroscope dynamic system consists of a 2-DoF drive mode oscillator along with 1-DoF sense-mode oscillator. Mass *m_2_* thus forms the passive mass of the 2-DoF drive mode oscillator and act as the vibration absorber of active mass *m_1_* [17].

**Figure 5:**
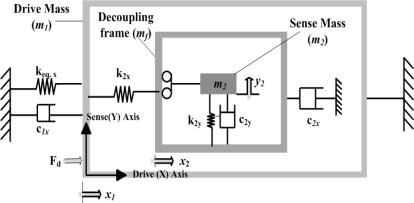
Lumped mass-spring model of the 3-DoF MVG.

Thus the equation of motion for active mass (*m_1_*), passive mass (*m_2_*+*m_f_*) and sensing element (*m*_2_) when subjected to an angular rate of Ω_z_ about the z-Axis (see Figure 5) become:

(1) $$\begin{array}{l} m_{1}{\ddot{x}}_{1}+c_{1x}{\dot{x}}_{1}+k_{\mathrm{eq}.x}\;x_{1}=k_{2x}\;(x_{2}-x_{1})+m_{1}\Omega_{z}{}^{2}\;x_{1}+F_{d}\;(t)\\ (m_{2}+m_{f}){\ddot{x}}_{2}+c_{2x}{\dot{x}}_{2}+k_{2x}\;(x_{2}-x_{1})=(m_{2}+m_{f})\Omega_{z}{}^{2}\;x_{2}\\ m_{2}{\ddot{y}}_{2}+c_{2y}\;{\dot{y}}_{2}+k_{2y}\;y_{2}=m_{2}\Omega_{z}{}^{2}\;y_{2}-2m_{2}\Omega_{z}{\dot{x}}_{2}-m_{2}{\dot{\Omega }}_{z}x_{2} \end{array}$$

where *m_f_* is the mass of the decoupling frame, *F_d_*(*t*) is the driving force applied to the active mass *m_1_* through the chevron-shaped thermal actuator at the driving frequency *ω_d_* and Ω*_z_* is the angular rate applied to the gyroscope about the z-axis. It is assumed that there is no anisoelasticity or anisodamping in the system. The Coriolis force that excites the mass *m*_2_ in the sense direction is 2*m*_2_Ω*_z_ẋ*_2_ and the Coriolis response of *m*_2_ in the sense direction (*y_2_*) provides a measure of the input angular rate.

### 2.2. Suspension Design Implementation:

Almost all existing micromachined vibratory gyroscopes operate on the principle of detection of rotation induced Coriolis force in the presence of an angular rate input. Therefore, the proof mass should be free to oscillate in two orthogonal directions, and constrained in other vibration modes. Thus, the suspension system design plays an important and critical role in achieving these objectives.

**Figure 6.**
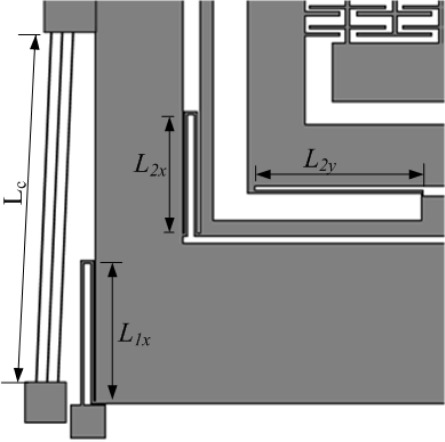
Suspension system configuration that forms the 2-DoF drive and 1-DoF sense mode oscillators.

The complete suspension system of the device is shown in Figure 6 and designed in such a way that the first mass *m_1_* with 1-DoF is fixed in the sense direction and free to oscillate in drive direction only. The second mass *m_2_* has 2-DoF; it is free to oscillate in both the drive and sense directions. Mass *m_2_* is nested inside a drive mode frame, the sense direction oscillations of the frame are constrained, where as the drive direction oscillations are forced to be in the designed drive direction. Thus the mass *m_2_* is free to oscillate only in the sense direction with respect to the frame, and the sense mode response will be orthogonal to the drive direction minimizing the anisoelasticities resulting in quadrature error.

The suspension connecting the mass *m_1_* with the substrate via anchors is comprised of four, double-folded flexures where each beam of length *L_1x_* in the folded flexure can be modeled as a fixed-guided beam deforming in the orthogonal direction to the axis of the beam, leading to an overall stiffness of:

(2) $$k_{1x}=\frac{4}{2}\;\left(\frac{12\mathrm{EI}}{L_{1x}{}^{3}}\right)=\frac{2{\mathrm{Etw}}^{3}}{L_{1x}{}^{3}}$$

Where *E* is the Young’s Modulus, *I=tw^3^/12* is the second moment of inertia of the rectangular beam cross section, *t* is the beam thickness and *w* is the beam width.

As the chevron-shaped actuator is directly attached to the mass *m_1_*, its stiffness will also affect the natural frequency of the mass *m_1_* in the drive direction. Neglecting the small angle of the chevron-shaped actuator, it can be modeled as fixed-fixed beam. For *N* beams of length *L_c_*, the stiffness of the chevron-shaped actuator can be calculated as [18]:

(3) $$k_{\mathrm{chev}}=N\;\left[\frac{192\mathrm{EI}}{L_{c}{}^{3}}\right]=N\;\left[\frac{16{\mathrm{Etw}}^{3}}{L_{c}{}^{3}}\right]$$

The total drive direction stiffness can be approximated by combining *k_1_x* and *k_chev_* as:

(4) $$k_{\mathrm{eq}.x}=\left[\frac{2{\mathrm{Etw}}^{3}}{L_{1x}{}^{3}}\right]+N\;\left[\frac{16{\mathrm{Etw}}^{3}}{L_{c}{}^{3}}\right]$$

The decoupling frame with mass *m_f_* is connected to mass *m_1_* via four double-folded flexures with a beam length of *L_2x_* which can be deformed in the drive direction resulting in the drive direction stiffness of:

(5) $$k_{2x}=\frac{4}{2}\;\left(\frac{12\mathrm{EI}}{L_{2x}{}^{3}}\right)=\frac{2{\mathrm{Etw}}^{3}}{L_{2x}{}^{3}}$$

The sensing mass *m_2_* is connected to the decoupling frame with four single folded flexures, each having a beam length of *L_2y_*. Since these sense mass flexures are stiff in the drive direction and deform only in sense direction, instability due to dynamic coupling between drive and sense modes is greatly reduced, minimizing zero rate drift of the gyroscope. The overall stiffness with a length of *L_2y_* for each beam is:

(6) $$k_{2y}=\frac{4}{1}\;\left(\frac{12\mathrm{EI}}{L_{2y}{}^{3}}\right)=\frac{4{\mathrm{Etw}}^{3}}{L_{2y}{}^{3}}$$

### 2.3. Damping Estimation:

The main energy dissipation in gyroscopes occurs due to the internal friction of the fluid confined between the proof mass and the stationary surfaces. The damping coefficients *c_1x_*, *c_2x_* and *c_2y_* in the gyroscope dynamic system shown in Figure 5 are mainly due to the viscosity of air between the masses and substrate. Assuming an instantaneously developed linear fluidic velocity profile, slide film damping for mass *m_1_* for the drive mode and mass *m_2_* for both drive and sense modes can be modeled as couette flow leading to:

(7) $$\begin{array}{l} c_{1x}=\mu_{e}\;\frac{A_{1}}{z_{0}}\\ c_{2x}=\mu_{e}\;\frac{A_{2}}{z_{0}}\\ c_{2y}=\mu_{e}\;\frac{A_{2}}{z_{0}} \end{array}$$

Where *A_1_* is the area of mass *m_1_* and *A_2_* is the area of mass *m_2_*, *z_0_* is the air gap between the proof masses and the substrate. The effective viscosity constant, *μ_c_* = *μ_p_p*, where *p* is the ambient pressure within the cavity of the packaged device, and *μ_p_* = 3.7 × 10^−4^*kg*/*m*^2^.*s.torr* is the viscosity constant for the air.

## 3. Prototype Modeling and Fabrication

### 3.1. Parametric optimization

Sense direction deflection of sense mass *m_2_* due to rotation induced Coriolis force is an important mechanical factor determining the performance of gyroscope. So the parameters of dynamical system must be optimized to maximize the sense direction amplitude of the mass *m_2_*.

For the parametric optimization of the dynamical system, 3-DoF gyroscope system has been decomposed into the 2-DoF drive and 1-DoF sense mode oscillator. Main objective of the parametric optimization in the 2-DoF drive mode is to maximize the rotation induced Coriolis force generated by mass *m_2_* to excite 1-DoF sense mode oscillator and is proportional to sensor sensitivity.

**Figure 7.**
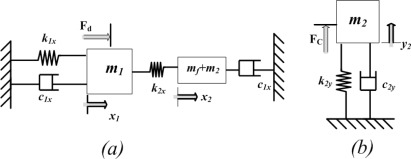
(a) Lumped mass-spring-damper model for 2-DoF drive mode oscillator (b) lumped mass-spring-damper model for 1-DoF sense mode oscillator of 3-DoF gyroscope.

2-DoF drive mode oscillator consists of drive mass *m_1_* (active mass). The sinusoidal force is applied to this mass by the chevron-shaped thermal actuator. The combination of frame and sense masses (*m_f_* + *m_2_*) comprises the vibration absorber (passive mass) of this 2-DoF oscillator. Approximating the gyroscope by a lumped mass-spring-damper model in Figure 7*(a)*, the equation of motion in the drive direction can be expressed as:

(8) $$\begin{array}{l} m_{1}{\ddot{x}}_{1}+c_{1x}\;{\dot{x}}_{1}+k_{1x}\;x_{1}=k_{2x}\;(x_{2}-x_{1})+F_{d}\;(t)\\ (m_{2}+m_{f}){\ddot{x}}_{2}+c_{2x}\;{\dot{x}}_{2}+k_{2x}\;(x_{2}-x_{1})=0 \end{array}$$

The equation of motion of the lumped mass-spring-damper model of the 1-DoF sense mode Figure 7*(b)* becomes:

(9) $$m_{2}{\ddot{y}}_{2}+c_{2y}\;{\dot{y}}_{2}+k_{2y}\;y_{2}=2m_{2}\Omega_{z}{\dot{x}}_{2}$$

When a constant-amplitude sinusoidal force *F_d_* = *F*_0_sin (*wt*) is applied on active mass *m_1_*, the steady state response of the 2-DoF system will be [17]:

(10) $$\begin{array}{l} X_{1}=\frac{F_{0}}{k_{1x}}\;\frac{1-\left(\frac{\omega }{\omega_{2x}}\right)+j\omega \;\frac{c_{2x}}{k_{2x}}}{\left[1+\frac{k_{2x}}{k_{1x}}-{\left(\frac{\omega }{\omega_{1x}}\right)}^{2}+j\omega \;\frac{c_{1x}}{k_{1x}}\right]\;\left[1-{\left(\frac{\omega }{\omega_{2x}}\right)}^{2}+j\omega \;\frac{c_{2x}}{k_{2x}}\right]-\frac{k_{2x}}{k_{1x}}}\\ X_{2}=\frac{F_{0}}{k_{1x}}\;\frac{1}{\left[1+\frac{k_{2x}}{k_{1x}}-{\left(\frac{\omega }{\omega_{1x}}\right)}^{2}+j\omega \;\frac{c_{1x}}{k_{1x}}\right]\;\left[1-{\left(\frac{\omega }{\omega_{2x}}\right)}^{2}+j\omega \;\frac{c_{2x}}{k_{2x}}\right]-\frac{k_{2x}}{k_{1x}}} \end{array}$$

where $\omega_{1x}=\sqrt{k_{1x}/m_{1}}$ and $\omega_{2x}=\sqrt{k_{2x}/(m_{f}+m_{2})}$ are the resonant frequencies of isolated active and passive mass-spring system, respectively. When the driving frequency, *ω_d_* = *ω*_2*x*_ the passive mass moves to exactly cancel out the applied input force *F_d_* on the active mass, and maximum dynamic amplification is achieved.

Maximization of the Coriolis force *F_c_* generated by mass *m_2_* requires a large proof mass *m_2_*, and large drive direction amplitude *x_2_*. However (*m_f_* + *m_2_*) should be minimized for high oscillation amplitudes of the passive mass if drive direction response of the passive mass is observed for varying *m_2_* values with *m_1_* being fixed. *ω*_2*x*_ is determined according to gyroscope operating frequency specifications, noting that larger Coriolis forces are induced at higher frequencies, but oscillation amplitudes become larger at lower frequencies [3].

As mass ratio *μ_x_* = (*m_f_* + *m*_2_)/*m*_1_ decreases, the resonant frequency separation of the 2-DoF drive mode oscillator decreases. However resonant frequencies should be far enough such that variations in the drive frequency away from *ω*_2*x*_ don’t cause significant changes in the passive mass amplitude [17]. Mechanical amplification depends upon the frequency ratio of isolated active and passive mass namely, *λ_x_* = *ω*_2*x*_/*ω*_1*x*_ which should be high enough for high mechanical amplification, and high oscillation amplitude of the passive mass [3].

### 3.2. Prototype Fabrication

A prototype 3-DoF gyroscope is designed to be fabricated by the Metal-Multi User MEMS Processes (MetalMUMPs) [20] for prototype verification of the design concept. MEMSPro is used for the designing mask layouts, design rule checks and process simulations for MetalMUMPs. MetalMUMPs is a low cost, commercially available, general purpose electroplated nickel micromachining process for MEMS devices available from MEMSCap. MetalMUMPs is a popular process for the fabrication of the poly/nickel powered gripper, the thermal-actuator based bistable micro-relay and MEMS variable capacitors.

**Figure 8.**
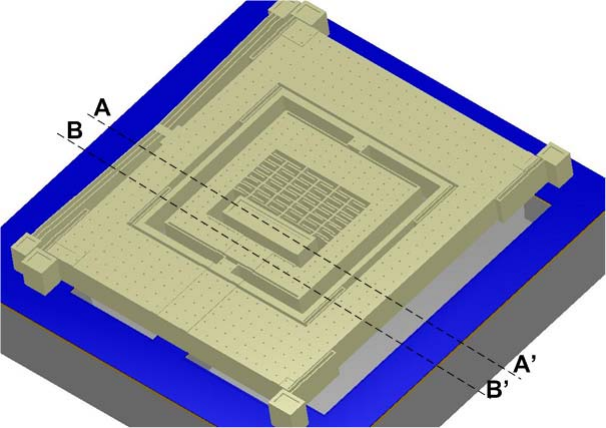
Microgyroscope fabricated through MetalMUMPs process using L-Edit of MEMSPro.

**Figure 9.**
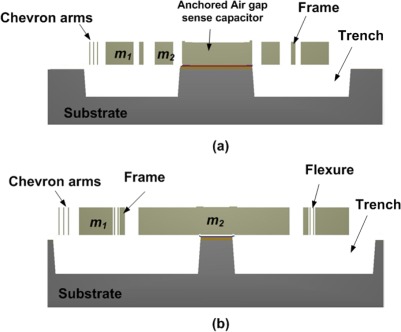
Cross sectional views of a microgyroscope (a) A-A’ and (b) B-B’.

This process consists of a 20 μm thick electroplated nickel layer used as the primary structural material and electrical interconnect layer. A doped polysilicon is also available for resistors, additional mechanical structures, and/or cross-over electrical routing. Two nitride layers (nitride 1 and nitride 2) are used as an electrical isolation layer whereas deposited oxide (PSG) is used for the sacrificial layers. A trench layer in the silicon substrate can also be incorporated for additional thermal and electrical isolation. The proposed microgyroscope model fabricated thru MetalMUMPs after process simulation in MEMSPro is shown in Figure 8. Cross sectional views are given in Figure 9*(a)–(b)* to illustrate different layer used during prototype fabrication.

**Figure 10.**
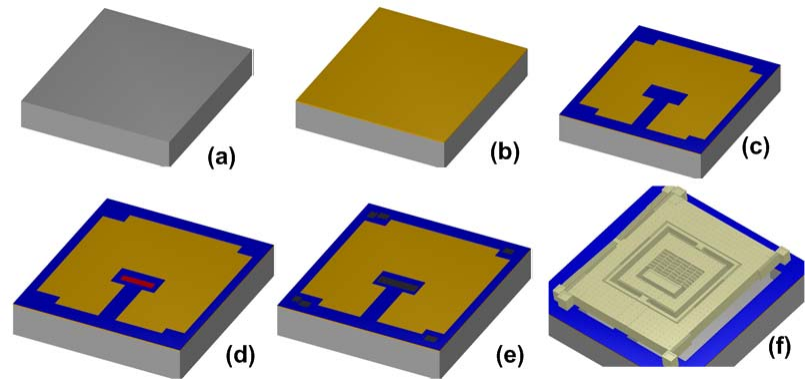
Process flow for the fabrication of microgyroscope using MetalMUMPs in MEMSPro (a) N-type 〈100〉 silicon wafer (b) 2 μm thick isolation oxide layer (c) patterning of 0.35 μm thick silicon nitride layers (d) patterning of 0.7 μm thick Polysilicon layer (e) patterning of anchor metal layer (f) patterning of 20 μm electroplated structural layer of Ni and trench etch in the substrate.

The process steps involved in the fabrication of the proposed MVG using MetalMUMPs are shown in Figure 10. A systematic description of all steps are given below:

1. MVG fabrication is started with an N-type 〈100〉 silicon wafer with a 2 μm thick silicon dioxide isolation layer.
2. A sacrificial Oxide 1 layer is 0.5 μm thick PSG used to define the trench below the moving parts of the MVG.
3. Two silicon nitride layers of thickness 0.35 μm are used for anchoring the fixed parts of the MVG to the substrate.
4. Polysilicon layer (Poly) is used to make cross-over electrical routing for the fixed part of the parallel plate air gap sense capacitor.
5. 1.1μm thick Oxide 2 layer of PSG is used to release the metal (Nickel) structure.
6. Thin layers of Cr and Pt is deposited at the anchoring locations by lift off technique following the deposition of a 500 nm Cu layer and a 50 nm Ti layer to form the plating base for Ni.
7. Thick photoresist is deposited to form the stencil for the electroplating. A 20 μm thick nickel layer is electroplated for the structural layer of the MVG.
8. Hole layer (HoleM) is added over the Metal to provide shorter release etch path under large metal feature.
9. Final steps are the release and silicon trench etch. The release is a series of wet chemical etches to first remove the Plating Base and then the sacrificial layers and the Isolation oxide layer over the trench areas. Finally, a wet chemical etch of the silicon, using KOH, is used to form a 25μm deep trench in the silicon substrate.

The overall size of the device is 2.2 mm × 2.6 mm. The movable parts of the MVG like the chevron-shaped actuator, proof masses and folded flexure are defined using the 20μm thick nickel layer. A 25μm deep trench is defined underneath the movable parts of the MVG to provide electrical and thermal isolation from the silicon substrate. The anchors and fixed parts are formed by the isolation oxide, nitride layers, anchor metal and nickel layers.

### 3.3. Design parameters

A dynamical system with the following parameter has been designed. The proof masses are *m*_1_ = 4.65 × 10^−7^*kg* and *m*_2_ = 1.18 × 10^−7^*k* and the decoupling frame mass is *m_f_* = 0.437 × 10^−7^*kg*. Thus for the drive mode oscillator, the active and passive proof mass values become *m*_1_*_x_* = 4.65 × 10^−7^*kg* and *m*_2*x*_ = 1.617 × 10^−7^*kg*. The spring constants calculated in drive direction for active and passive mass are *k_eq.x_* = 239.39 *N*/*m* and *k*_2*x*_ = 54.37 *N*/*m*, respectively, whereas the spring constant for suspension of the sensing mass in the sense direction is *k*_2*y*_ = 61.12 *N*/*m*. These spring constants are calculated by finite element method (FEM) using Thermoelectromechanical (TEM) Analysis module of MEMS Design software IntelliSuite.

In the drive mode, the resonant frequencies of the isolated active and passive mass-spring systems are $\omega_{1x}=\sqrt{k_{\mathrm{eq}.x}/m_{1x}}=3.61\mathrm{kHz}$ and $\omega_{2x}=\sqrt{k_{2x}/m_{2x})}=2.92\mathrm{kHz}$, respectively, whereas the resonant frequency of the isolated sense mass *m_2_* in the sense direction is $\omega_{2y}=\sqrt{k_{2y}/m_{2}}=3.62\mathrm{kHz}$. The resulting frequency ratio hence is *λ_x_* = *ω*_2*x*_/*ω*_1*x*_ = 0.81 and the mass ratio of *μ_x_* = (*m_f_* + *m*_2_)/*m*_1_ = 0.347. With these parameters the location of the two expected resonance peaks in the drive mode frequency response were calculated as *ω*_*x*−*n*_1__ = 2.45*kHz* and *ω*_*x*−*n*_2__ = 4.30*kHz* based on the relation [17]:

(11) $$\begin{array}{l} \omega_{x-n_{1}}=\sqrt{1/2(1+\mu_{x}+1/\lambda_{x}{}^{2}-\sqrt{{\left(1+\mu_{x}+1/\lambda_{x}{}^{2}\right)}^{2}-4/\lambda_{x}{}^{2})}}\Omega_{2x}\\ \omega_{x-n_{2}}=\sqrt{1/2(1+\mu_{x}+1/\lambda_{x}{}^{2}+\sqrt{{\left(1+\mu_{x}+1/\lambda_{x}{}^{2}\right)}^{2}-4/\lambda_{x}{}^{2})}}\Omega_{2x} \end{array}$$

The chevron-shaped thermal actuator has six 1120 μm (*L_c_*) hot arms with 60 μm wide center section which is attached with the active mass *m_1_* for a total length of 2300 μm from anchor to anchor. These hot arms are 8 μm wide and overall thickness of the device is 20 μm made from electroplated Ni. Referring to Figures 2*(a)* and 4*(b)*, the calculated angle *θ* for the chevron-shaped actuator is 1.78°.

## 4. FEM Methodology

In this section, device level FEM simulation methodology for microgyroscope using Finite Element Analysis (FEA) is proposed. For this purpose thermoelectromechanical (TEM) analysis module of the MEMS Design software IntelliSuite is used. These TEM analyses will verify the design concept and parameters, discussed in design implementation section.

**Figure 11.**
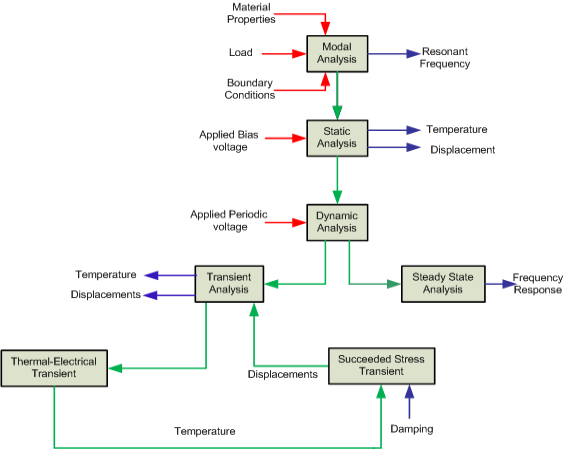
FEM methodology for sequential thermoelectromechanical analysis. Red arrows show the input parameters whereas blue arrows show the output of the respective analysis.

Before starting FEA, the drafting module of IntelliSuite called 3D Builder is used to build and mesh the three-dimensional geometry of the MEMS structure. Then this model is transferred to TEM module where user assigns material properties, loads and boundaries to fully analyze a device in the static, frequency and dynamic domain. The FEM based sequential TEM analysis methodology for the proposed device has been illustrated in Figure 11. During the implementation of this methodology, simpler and faster simulations are performed prior to lengthy dynamic simulations to understand the initial behavior of the device.

We started from modal analysis to calculate resonant frequencies and mode shape followed by static analysis for temperature and displacement distribution. Afterwards dynamic simulations have been performed. These simulations include steady state as well as transient analyses to get frequency responses in the drive and sense directions along with their stress distributions when a dynamic voltage is applied upon the device.

### 4.1. Modal Analysis

Modal analysis was performed to verify the natural frequencies and their respective mode shape for the proposed gyroscope. In MetalMUMPs, the structural layer of electroplated nickel has a residual stress of 100 MPa [20]. Therefore, while calculating natural frequencies and associated mode shapes, this value of residual stress has been incorporated along with other thermophysical properties of nickel (Ni) for accurate results. A nonlinear analysis assumption results in nonlinear stiffness terms of the system which modifies the stiffness matrix. This modified stiffness matrix is used to solve the eigenvalue problem to obtain the natural frequencies.

**Figure 12.**
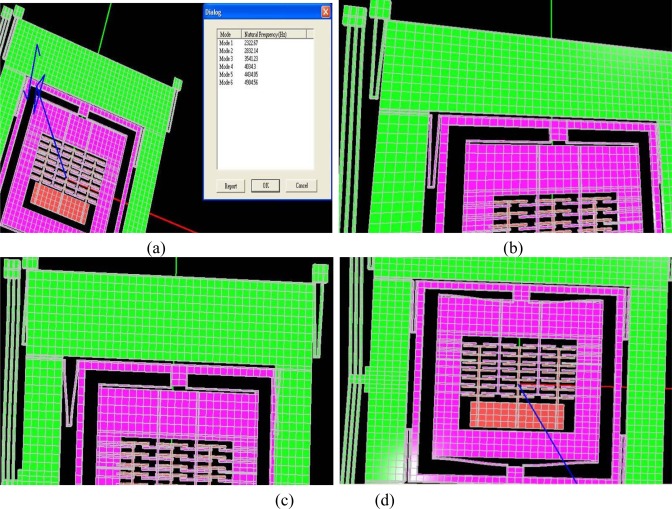
(a) Modal analysis results showing resonant frequencies. For better visualization of these oscillations, modal animations were carried out at a scale factor of 100 (b) first drive direction mode *ω*_1*x*_ at 2.32 kHz (c) Second drive mode *ω*_2*x*_ at 4.43 kHz and (d) sense mode *ω*_2*y*_ at 3.54 kHz.

The predicted resonant frequencies from the modal analysis of the device are listed in Table 1 and their associated mode shapes are shown in Figures 12(a)–(c). Two resonance frequencies for the 2-DoF drive mode oscillator are observed at 2.32 kHz and 4.43 kHz, which compares well to the analytically calculated values of 2.45 kHz and 4.30 kHz in Section 3.3. The sense mode frequency of 3.54 kHz is located inside the drive mode flat region as desired, allowing the gyroscope to be operated at resonance in the sense mode and within the flat region in the drive mode.

One of the important factors affecting the results of FEA is meshing. An optimized mesh size generates FEA results with desired accuracy in a reasonable time. Mesh size optimization for the model was carried by performing modal analysis at different mesh sizes and compared the subsequent results. These analyses showed that changing the mesh size from 100 to 50 μm, the variation in resonant frequency was less 2%. However the computational time required for simulation with 50 μm mesh size was six times than that with 100 μm mesh size. This computational time increased exponentially for more complicated analyses like dynamic analysis. Based on these observations, a mesh size of 100 μm was chosen for performing FEA simulations for 8-node brick element. This mesh size created 13419 total nodes for the model.

### 4.2. Static Analysis

Applied loads and the boundary conditions are the major factors influencing device performance in addition to the material properties (residual stress, its gradient, density, resistivity etc.). Static analysis is performed to analyze the device initial response to these parameters which calculates the results for the stress distribution, displacement distribution and mechanical deformation of the structure. For the thermally actuated device, static analysis comprises of thermal-electrical and thermal-stress analyses. Thermal-electrical analysis solves coupled thermal electrical equations. The coupling arises from the internal heat generation in the device, which is a function of electrical current density. Thermal-stress analysis calculates mechanical stresses in the device due to the thermal loads calculated during the thermal-electrical analysis.

The proposed device has been modeled as a heated horizontal plate that convects heat from top and bottom faces. Radiation losses were ignored due to the fact that overall temperature achieved by the chevron-shaped actuator against the applied voltage was not high enough. Since area to length ratio of the chevron-shaped actuator is small which minimizes the heat loss by conduction mechanism. The thermal model is implemented by specifying/assuming an overall convective heat transfer coefficient (film coefficient) for the bottom and top faces of the device along with the substrate temperature which acts as a heat sink. This approach is computationally more efficient than meshing the substrate of the device [21].

**Figure 13.**
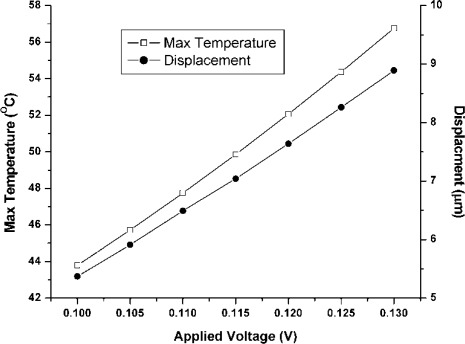
Drive direction displacement and maximum temperature when a static voltage is applied.

**Figure 14.**
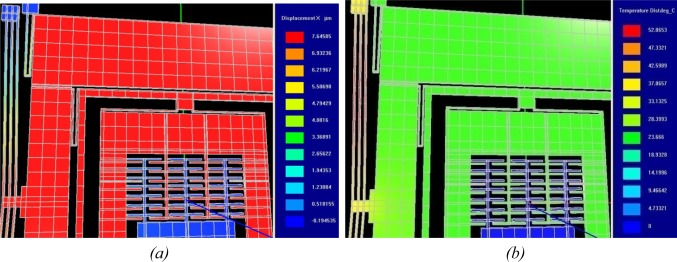
(a) Shows the drive displacement of 7.64 μm and (b) shows the temperature of 52.06 °C achieved by the device at 0.12 Vdc.

Predicted displacement vs. voltage and temperature vs. voltage plots are presented in Figure 13. This analysis is carried out over a range of applied static voltages from 0.10–0.13V with an increment of 0.05V. As the temperature rise during this voltage change is small, so change in resistivity of nickel with temperature has been ignored in these analyses. The achieved displacement and temperature are the function of the V^2^ at constant resistivity. A drive displacement of 115 μm/V along with a temperature rise of 422 °C /V predicts that voltage-stroke ratio of the proposed device is very high. In comparison with electrostatic comb drives, the applied static voltage may be in the range of 20V or higher to achieve drive displacement amplitude of approximately 5μm [22, 25]. Using the chevron-shaped thermal actuators we can achieve a similar displacement with an applied voltage of 0.1V. Displacement and temperature profile of the device at 0.12V DC is shown in Figure 14(a)–(b). A predicted displacement of 7.64 μm with a temperature of 52.06 °/C is achieved by the device at this voltage.

### 4.3. Dynamic Analysis

For the proposed chevron-based gyroscope, an initial dynamic thermal-electrical transient analysis was performed to solve the coupled thermal electrical equations. This analysis is similar to the static analysis; the only difference being that a periodic voltage instead of DC bias was used.

The design approach as described in Section 2.1 implies to operate the gyroscope at the sense direction resonance frequency that is 3.54 kHz. Consequently sense mass will attain the maximum possible amplitude in sense direction in response to the rotation induced Coriolis force. With a periodic signal input, the chevron-shaped actuator will heat and cool cyclically. The power delivered to the actuator is independent of the voltage polarity, one electrical cycle results in two mechanical cycles [6]. This means that applied actuation voltage frequency will be half of device operating frequency. So these dynamic analyses are carried out at voltage amplitude of 0.5V applied at a frequency of 1.77 kHz which is half of the device’s operating frequency due to the reason mentioned above.

Sampling rate is another important factor for transient analysis which effects the computational time like meshing. A sampling rate of 10 points per cycle is considered sufficient for an initial transient simulation [19]. For the proposed gyroscope, time required to complete one cycle is 282 μsec. We performed transient analysis for 500 μsec to examine two initial cycles of the proposed device.

**Figure 15.**
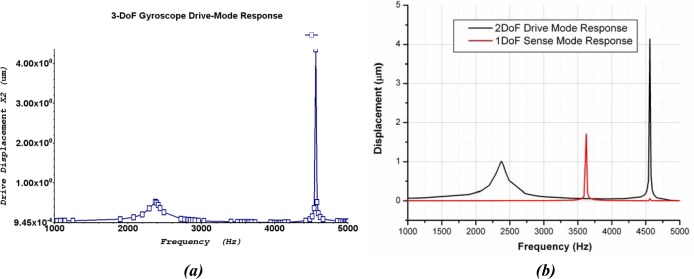
(a) 2-DoF Drive mode oscillator frequency response: showing the 1^st^ and 2^nd^ resonant frequency peak at 2.32 kHz and 4.43 kHz, respectively. (b) Combined response of 3-DoF gyroscope with 2-DoF Drive and 1-DoF sense-mode oscillator demonstrating that the sense mode resonant frequency is located inside the drive-mode flat region.

The frequency response shown in Figure 15*(a)* predicts that the 2-DoF drive-mode oscillator has two resonant peaks located at the 2.32 kHz and 4.43 kHz, which result in a wider bandwidth of 2.11 kHz. The 1-DoF sense mode oscillator has a single peak located at 3.62 kHz. The combined frequency response of the 2-DoF drive and 1-DoF sense-mode oscillators demonstrating that the sense mode resonant frequency is located inside the drive-mode flat region is shown in Figure 15*(b)*. The flat region of 2-DoF drive mode oscillator having wider bandwidth can be precisely overlapped with the resonance peak of the 1-DoF sense mode oscillator without feedback control.

In a 3-DoF micromachined gyroscope system *m*_2_ forms the passive mass of the 2-DoF drive mass oscillator and acts as a vibration absorber of the driven mass *m*_1_. While absorbing the oscillations of the active mass, passive mass itself achieves much larger drive amplitude than driven mass *m*_1._ Thus the actuation range of the chevron-shaped thermal actuator attached to the driven mass *m*_1_ is narrow. This makes the actuator more stable and linear in its response. While the sensing mass element *m*_2_ oscillates with larger drive amplitude, it induces a significant Coriolis force in response to rotation. This improves the sensitivity of the device due to larger amplitude in the sense direction.

**Figure 16.**
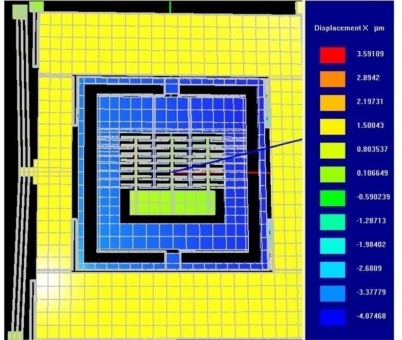
Displacement profile of the 3-DoF micromachined vibratory gyroscope excited by a sinusoidal voltage of 0.5V amplitude and a frequency of 1.77 kHz. Due to mechanical amplification, the passive mass *m_f_* + *m*_2_ (blue) achieves a displacement of 4.07μm keeping the active mass *m*_1_ (yellow) at a displacement of 1.5 μm.

**Figure 17.**
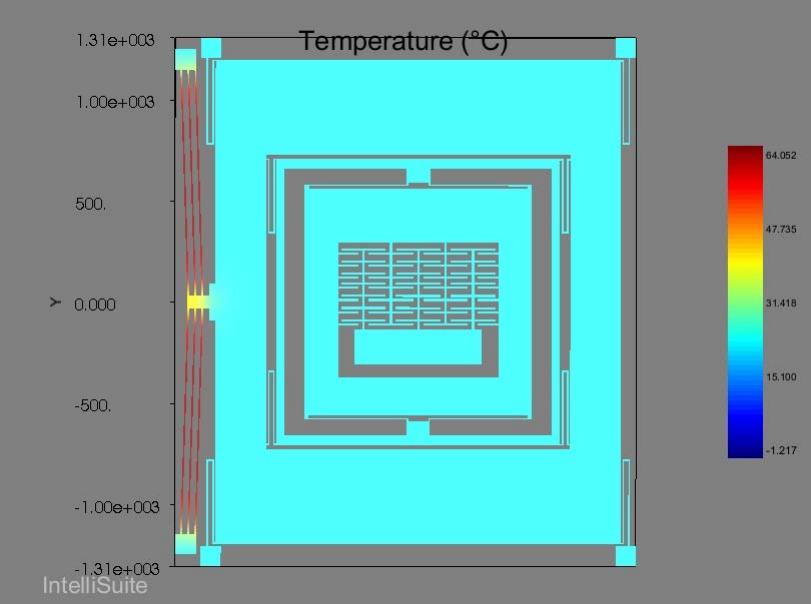
Temperature profile of the 3-DoF micromachined vibratory gyroscope when excited by a sinusoidal voltage of 0.5V amplitude and a frequency of 1.77 kHz.

FEA based dynamic simulation results are shown in Figure 16. The thermal actuator was driven by a sinusoidal voltage of 0.5V and 1.77 kHz; this results in a motion of thermal actuator of twice the frequency. The predicted drive displacement achieved by the passive mass is 4.1μm (shown blue in Figure 16), whereas the oscillation amplitudes of the active mass is 1.5μm (shown yellow in Figure 16). This demonstrates mechanical amplification of the oscillation amplitude of the sensing element *m_2_* in the drive direction. This causes a large Coriolis force generation increasing the sensitivity of the gyroscope. The temperature profile developed across the device when subjected to the same voltage excitation signal is shown in Figure 17.

### 4.4. Coriolis Response:

In order to attain the maximum possible oscillation amplitudes in response to a Coriolis force, the design concept suggests that gyroscope should be operated at the sense direction resonance frequency of mass *m*_2_.

**Figure 18.**
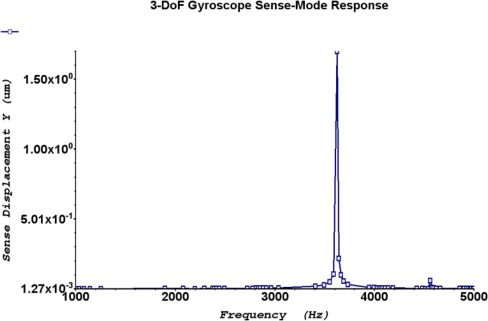
1-DoF Sense mode oscillator frequency response when 1 °/s rotation rate is applied along with 0.5V AC. Resonance peak at 3.54 kHz with an amplitude of 1.70 μm is in close agreement with the analytically estimated sense displacement due to rotation induced Coriolis force.

Results in Section 4.3 show drive amplitude of 4.1μm is achieved by the drive-mode passive mass when the device is operated at a sinusoidal voltage of 0.5V at a frequency of 1.77 kHz. Incorporating an angular rate of 1 °*/s* along the z-axis, the sense mass *m_2_* of 3-DoF gyroscope achieves the amplitude of 1.70 μm as shown in Figure 18. Coriolis force can be analytically calculated to verify simulated sense amplitude of mass *m_2_* using expression *F_c_* = 2*m*Ω*v*. From this expression Coriolis force of 0.011 *μN* is obtained at an angular rate of 1 °*/s*. Assuming that the microgyroscope is vacuum packaged with a quality factor of 10,000 in sense direction “*y*”, the sense mass will achieve a displacement of 1.76 *μm* in sense direction in response to the applied Coriolis force from the expression in Equation 12.

(12) $$y_{2}=\frac{Q_{y}\times F_{c}}{k_{y}}$$

This analytically predicted sense displacement is in close agreement with simulated oscillation amplitude of 1.70μm achieved by the sense mass in response to the Coriolis force. Figure 18 shows the frequency response of the 1-DoF sense mode oscillator when a rate of 1 °/s applied to microgyroscope. This sense direction oscillation amplitude results in a mechanical sensitivity of 1.7μm/°/s at a voltage of 0.5V.

### 4.5. Capacitive detection using parallel plates

**Figure 19.**
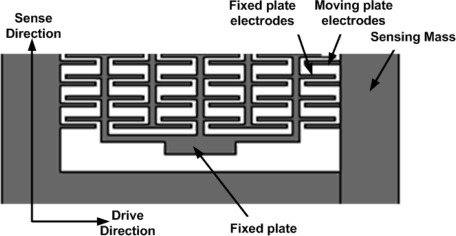
Capacitive detection mechanism using parallel plates

In our proposed design, we used parallel plate sense capacitors attached to the sense mass to detect the deflection in the sense direction in response to the Coriolis force. This deflection can be sensed as a difference in capacitance. The mass deflection changes the electrode gap and resulting capacitance change is detected. In case of positive displacement, the plates attached to the sensing mass approached near the fixed plate electrodes increasing its capacitance as shown in Figure 19. A standard differential capacitance bridge can be used to translate this deflection to a voltage.

### 4.6. Estimated Power consumption

In MEMS, power consumption is generally a great concern. Normally the heat actuators consume more power than the other type of the actuators including electrostatic one. Generally polysilicon films are used to make such thermal actuators which need high operating temperatures to produce sufficient force and displacement owing to the small thermal expansion coefficient of Si. On the other hand, metal based thermal actuators can be operated at much lower temperature owing to their much larger thermal expansion coefficient.

The power consumed by the single arm of the chevron-shaped thermal actuator may be estimated by the expression given in Equation 13. As the chevron-shaped actuator attached to our proposed model, consists of three hot arms so the final power consumption can be approximated by multiplying this expression by 3.

(13) $$P=\frac{V^{2}\;A}{\rho_{\mathrm{Ni}}\;L}$$

where *V* is the applied voltage across the chevron-shaped actuator, *A* is the cross sectional area of the actuator arm, *ρ_Ni_* is the resistivity of the Nickel (Ni) and *L* is the anchor to anchor length of the chevron-shaped actuator. Using the simple trigonometric expression, the calculated anchor to anchor arm length of the chevron-shaped actuator is 2300 μm which is 20 μm thick and 8 μm wide. Resistivity is an important parameter in the development of the MEMS based heat actuators. The resistivity of the electroplated Ni (20 × 10^−8^Ω*m*) doesn’t change significantly with the plating conditions. The average resistivity of the plated Ni films is about three times that of the bulk value [23]. As major temperature change occurs at the hot arms of the chevron-shaped actuator as shown in Figure 17, so we ignored the rest of the device geometry for the power estimation. So using the expression given in Equation 13, the estimated power consumption of our device is 0.26 Watts which is reasonably good in comparison to the microgyroscope using actuator other than the heat actuators.

## 5. Discussion

For the last couple of years, MEMS based thermal actuators have been emerged as one of the most important MEMS devices, which are able to deliver a large force with large displacement. They are being successfully used for various applications in electro-optical-communication, micro-assembly and micro-tools. Currently Si-based materials have been predominantly used to fabricate thermal actuators due to its mature process and stress-free materials.

We have proposed a microgyroscope model with a silicon substrate and electroplated Ni as a structural material using the MetalMUMPs. Thermal actuators based on metal materials generally have a number of advantages over Si-based ones due to their large thermal expansion coefficients, thus they can deliver large displacements and forces and consumes less power, and therefore they are much more efficient than Si-based ones. In such thermal actuators, cooling rate is a great concern, which limits the bandwidth and amplitude of the actuator. At DC voltage or in case of AC voltage at low frequencies the actuator reaches at high temperature, thus producing higher displacements. As we increase the frequency of the applied AC voltage, the actuator keep on oscillating, however its sinusoidal amplitude decreases. At high frequencies, the actuator cannot heat or cool fast enough and it deflects to a fixed position equal to the equivalent RMS value of the signal. This is the reasons that our static simulations are predicting more resultant displacements at less DC voltages then that of the displacements predicted by the dynamic analysis at relatively higher AC voltage. Furthermore, as we are using mechanical amplification in the drive mode which works only for an AC excitation, but not for a static voltage so therefore, the DC and AC case are indeed difficult to compare in terms of achievable displacements. As the operating frequency of the device is constant, so to achieve displacements similar to DC analysis, applied AC voltage will have to increase which will results into the increased device’s power consumption.

When proposing this model, two cares has been taken into consideration. Firstly, the operating frequency of the proposed microgyroscope shouldn’t be high enough to effect its drive mode vibration displacement due to the cooling problem. Secondly, higher mechanical sensitivity of 1.7μm/°/s in response to the Coriolis force at an angular rate of 1 °/s may cause pull-in and non linearity in the device response. So to avoid this issue, initial gap among the parallel plates of the sensing electrodes is kept high. In [24]:

(14) $$G_{P-I}=\frac{2}{3}\;G_{0}$$

where *G_P-I_* is the pull in gap and *G_0_* is the initial gap. The initial gap between the parallel plates, designed to sense Coriolis response, is kept 10 μm. This gap is high enough to avoid this pull in effect at this high sensitivity.

Motion stability issue of thermal actuator has been addressed by many researchers in the past. A detailed mechanical motion analysis of the Nickel based chevron-shaped thermal actuator has been done by Enikov *et al*. in [10], in which they developed experimental and numerical displacement model for the chevron-shaped thermal actuator to account for the lateral deflection due to both thermal axial load and transversely applied external load. Stevenson and Lai have demonstrated experimentally that micro-electro-thermal actuators are able to provide stable driving motion at kHz level [25]. They developed a microgripper, driven by a chevron-shaped actuator and successfully operated it at the frequency of 33.9 kHz applying 1V ac signal. Since the operating frequency of our proposed device is 3.54 kHz which is almost 10 times less than the above mentioned device so we don’t predict any motion instability issue with our proposed gyroscope model.

As far as velocity stability of micromachined gyroscope is concerned, it is indeed an important point to be considered. Our present design works on an open loop drive actuation mechanism. In a future design, similar techniques for drive velocity stabilization as for electrostatic gyroscopes could be incorporated. For example, if capacitive comb fingers were added to the drive mass, its position could be dynamically measured, and a feedback loop to the excitation voltage could be constructed, using automatic gain control and PLL control schemes.

To avoid non-linear behavior of the device, the operating range of the chevron-shaped actuator is kept very low using the dynamic amplification. Due to amplification, the passive mass of the 2-DoF drive mode oscillator achieves larger displacement in drive direction without considerable movement of the active mass, directly attached with the actuator. Thus the actuation range of the chevron-shaped thermal actuator attached to the active mass remains narrow. This makes the actuator more stable and linear in its response.

In the end a comparison of the proposed gyroscope with some existing model with comb drive based electrostatic actuation, developed by Alper *et al.* at Middle East Technical University (METU, Turkey) [26] and by Acar *et al*. at University of California, Irvine (UCI, USA) [27] is given in Table 2.

## 6. Conclusions

This paper presents a novel design concept of a thermally actuated Ni based non-conventional 3-DoF micromachined gyroscope utilizing a chevron-shaped thermal actuator to drive proof mass. The results demonstrate a new paradigm in MEMS gyroscope design where the chevron-shaped thermal actuator has successfully shown its potential to replace the traditional interdigitated comb drive electrostatic actuator. Exploiting the thermophysical properties of electroplated Ni, high drive direction amplitudes of 5.38 μm and 4.1 μm at low actuation voltage of 0.1 V_DC_ and 0.5 V_AC_ respectively was predicted with reduced device size of 2.2 × 2.6 mm^2^. This study further shows that a low cost commercially available MetalMUMPs can be a cost effective fabrication option for the microgyroscopes. The predicted mechanical sensitivity of the proposed design is 1.7 μm/°/s in the vacuum with an estimated power consumption of 0.26 watts. The wide bandwidth frequency response of 2.11 kHz is attained using 2-DoF Drive and 1-DoF sense mode oscillator approach, thus improving device robustness achieved by the mechanical system design. The stability of the device is ensured by keeping the actuation range of the chevron-shaped thermal actuator is narrow. A comprehensive description of FEA methodology to perform sequential thermoelectromechanical analysis in IntelliSuite has also been covered in this study. The MEMS gyroscopes of this class are expected to yield reliable, robust, low cost and high performance vibratory rate gyroscope for high volume applications.

## Figures and Tables

**Figure 3.**
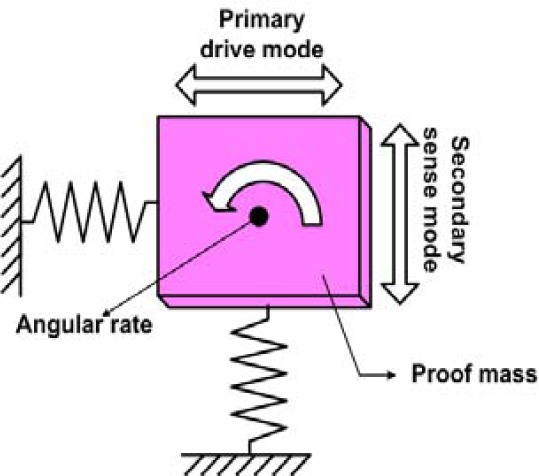
Operating principle of resonant micromachined vibratory gyroscope [12].

**Table 1.** Comparison of Simulated and analytical results for natural frequencies for drive and sense modes.

**Mode No**	**Simulated Resonant Frequency (kHz)**	**Analytical Resonant Frequency (kHz)**	**Remarks**

1	2.32	2.45	1^st^ Drive Mode *ω*_1*x*_
3	3.54	3.62	1^st^ sense Mode *ω*_2*y*_
5	4.43	4.30	2^nd^ Drive Mode *ω*_2*x*_

**Table 2.** A comparison of the proposed gyroscope with some existing models developed by Alper *et al*. [26] and Acar *et al*. [27].

**Institute/University**	**METU**	**UCI**	**PIEAS/GIKI**

**Type**	Resonant	Non-resonant	Non-resonant
**Material**	Nickel	Polysilicon	Nickel
**Fabrication Process**	DRIE	DRIE	MetalMUMPs
**Size**	2.9 × 2.9 mm^2^	4.0 × 4.0 mm^2^	2.2 × 2.6 mm^2^
**Structural layer thickness**	18 μm	100 μm	20 μm
**Operating Frequency**	4090 Hz	752 Hz	3540 Hz
**Applied Voltage**	Not available	25V_DC_ +3V_AC_	0.5V_AC_
**Drive Displacement**	10 μm	5.8 μm	4.1 μm
